# Supplementary material for: Impact of Reinfection with SARS-CoV-2 Omicron Variants in Previously Infected Hamsters
Source: J Virol. 2023 Jan 12;97(1):e01366-22. doi: 10.1128/jvi.01366-22 (PMC9888231; doi:10.1128/jvi.01366-22)
Supplement: Supplemental file 1 — Tables S1 to S6. Download jvi.01366-22-s0001.pdf, PDF file, 0.1 MB [file jvi.01366-22-s0001.pdf]

Impact of reinfection with SARS-CoV-2 Omicron variants in previously infected hamsters

Nozomi Shiwa-Sudo,[a] Yusuke Sakai,[a] Naoko Iwata-Yoshikawa,[a] Shinji Watanabe,[b] Souichi Yamada,[c] Yudai Kuroda,[d] Tsukasa Yamamoto,[d] Masayuki Shirakura,[b] Seiichiro Fujisaki,[b] Kaya Miyazaki,[b] Hideka Miura,[b] Shiho Nagata,[b] Shuetsu Fukushi,[c] Ken Maeda,[d] Hideki Hasegawa,[b] Tadaki Suzuki,[a] Noriyo Nagata[a]#

[a]Department of Pathology, National Institute of Infectious Diseases, Tokyo, Japan.

[b]Research Center for Influenza and Respiratory Viruses, National Institute of Infectious Diseases, Tokyo, Japan.

[c]Department of Virology I, National Institute of Infectious Diseases, Tokyo, Japan.

[d]Department of Veterinary Science, National Institute of Infectious Diseases, Tokyo, Japan.

Running Head: Reinfection of SARS-CoV-2 variant in a hamster model

#Address correspondence to Noriyo Nagata, nnagata@niid.go.jp

SUPPLEMENTARY MATERIAL

Supplementary Table 1 Omicron BA.1 variants detected in nasal wash fluid samples from hamsters after second inoculation

Variant			Group/Animal No.																						
			DMEM-BA1				A-BA.1			B.1.1.7-BA.1				B.1.351-BA.1				B.1.617.2-BA.1				B.1.1.529, BA.1-BA.1			
Nuc no. in Wuhan	AA substitution	protein	#1	#2	#3	#4	#5	#6	#7	#9	#10	#11	#12	#13	#14	#15	#16	#17	#18	#19	#20	#21	#22	#23	#24
209T	Silent	-						Y<br>C:44.2%																	
804G	ORF1a: G180E	nsp1:G180E		R<br>A:12.8%																					
921T	ORF1a: L219Q	nsp2:L39Q												W<br>A:33.1%											
1915C	Silent	nsp2								Y<br>T:13.0%															
7051C	Silent	PLpro (nsp3)							Y<br>T:24.9%																
7945C	Silent	PLpro (nsp3)											Y<br>T:33.3%												
9451C	Silent	nsp4												Y<br>T:17.3%											
9970A	Silent	nsp4								R<br>G:45.1%															
10934T	ORF1a: F3557L	3CL (nsp5): F294L						Y<br>C:37.6																	
11020C	Silent	nsp6							Y<br>T:28.7%																
11083G	ORF1a: L3606F	nsp6:L37F																				K<br>T:11.4%			
11355C	ORF1a: A3697V	nsp6:A128V													Y<br>T:33.0%										
11674C	Silent	nsp6									Y<br>T:21.5%														
11750C	ORF1a: L3829F	nsp6:L260F	Y<br>T:15.5%	Y<br>T:36.4%	Y<br>T:12.5%		Y<br>T:13.7%		Y<br>T:39.4%		Y<br>T:51.3%							Y<br>T:45.4%			Y<br>T:10.5%	Y<br>T:12.0%		Y<br>T:19.3%	
12747C	ORF1a: T4161I	nsp9:T21I								Y<br>T:13.6%						Y<br>T:23.6%									
12884A	ORF1a: T4207A	nsp9:T67A																	R<br>G:20.6%		R<br>G:10.5%		R<br>G:73.1%		
13131A	ORF1a: Q4289R	nsp10:Q36R																						R<br>G:35.2%	
15211A	ORF1b: T582A	RdRP (nsp12): T591A											R<br>G:10.5%								R<br>G:36.6%				
19206C	Silent	nsp14																						Y<br>T:28.9%	
20486A	ORF1b: K2340T	nsp15:K289T	M<br>C:39.6%	M<br>C:35.8%	M<br>C:46.3%	M<br>C:40.9%	M<br>C:38.4%	M<br>C:12.0%	M<br>C:77.2%		M<br>C:30.1%	C<br>C:95.6%	M<br>C:26.5%		M<br>C:46.8%	M<br>C:24.2%	M<br>C:69.1%	M<br>C:24.5%		C<br>100%	M<br>C:16.4%	M<br>C:50.8%		M<br>C:24.0%	
21561C	Silent	-																					Y<br>T:10.2%		
21562A	Silent	-																					W<br>T:10.2%		
21565G	S: M1 del	S: M1 del																					R<br>A:10.1%		
21925T	Silent	S												Y<br>C:56.7%											
22151A	S: I197L	S: I197L						M<br>C:40.9%																	
22194A	S: N211del, L212I	S:N211I, L212del					A/del<br>A:15.1%																		
22195T							T/del<br>T:15.1%																		
22196T							T/del<br>T:15.1%																		
23788T	Silent	S																						Y<br>C:34.9%	
28971A	N: K233R	N: K233R																			R<br>G:12.1%				
29645G	ORF10: V30L	ORF10: V30L	K<br>G:28.4%	K<br>G:23.1%	K<br>G:22.1%	K<br>G:22.4%	K<br>T:74.2%			K<br>T:16.8%	K<br>T:78.1%					K<br>G:50.3%		K<br>G:13.3%	K<br>G:21.5%		K<br>G:21.6%		K<br>G:10.7%	K<br>G:50.3%	
PANGO			BA.1.18	BA.1.18	BA.1.18	BA.1.18	BA.1.18	BA.1.18	BA.1.18	BA.1.18	BA.1.18	BA.1.18	BA.1.18	BA.1.18	BA.1.18	BA.1.18	BA.1.18	BA.1.18	BA.1.18	BA.1.18	BA.1.18	BA.1.18	BA.1.18	BA.1.18	BA.1.18

Supplementary Table 2 Omicron BA.1 variants detected in nasal wash fluid samples from hamsters after second inoculation

Variant			Inoculum		Group/Animal No.				Inoculum		Group/Animal No.			
			1st	2nd	DMEM-BA.1				1st	2nd	BA.1-BA.1			
Nuc no. in Wuhan	AA substitution	protein	DMEM	TY38-873	#1	#2	#3	#4	TY38-873	TY38-873	#17	#18	#19	#20
11048G	ORF1a: V3595F	nsp6:V26F		G					G	G				K
														T:11.1%
11750C	ORF1a: L3829F	nsp6:L260F		C	Y			Y	C	C		Y		
					T:12.9%			T:15.9%				T:37.7%		
12747C	ORF1a: T4161I	nsp9:T21I		C					C	C	Y	Y		
											T:17.8%	T:13.0%		
13131A	ORF1a: Q4289R	nsp10:Q36R		A					A	A	R			
											G:29.0%			
20486A	ORF1b: K2340T	nsp15:K289T		A	M	M	M	M	A	A	M	M	M	M
					C:46.5%	C:45.0%	C:49.9%	C:46.1%			C:34.5%	C:24.3%	C:89.8%	C:44.7%
25487C	ORF3a: T32I	ORF3a:T32I		C					C	C		Y		
												T:11.8%		
26263G	E: E7K	E: E7K		G					G	G		R		
												A:10.4%		
28391C	N: R40C	N: R40C		C					C	C	Y			
											T:23.1%			
29645G	ORF10: V30L	ORF10: V30L		T	K	K	K	K	T	T	K	K		K
					G:19.3%	G:22.5%	G:16.2%	G:19.6%			G:32.6%	G:52.8%		G:30.0%
PANGO				BA.1.18	BA.1.18	BA.1.18	BA.1.18	BA.1.18	BA.1.18	BA.1.18	BA.1.18	BA.1.18	BA.1.18	BA.1.18

Supplementary Table 3 Omicron BA.1 variants detected in nasal wash fluid samples from hamsters after second inoculation

Variant			Inoculum		Group/Animal No.				Inoculum		Group/Animal No.			
			1st	2nd	DMEM-BA.1.1				1st	2nd	BA.1-BA.1.1			
Nuc no. in Wuhan	AA substitution	protein	DMEM	TY38-871	#5	#6	#7	#8	TY38-873	TY38-871	#21	#22	#23	#24
9005G	ORF1a: V2914I	nsp4:V151I		G					G	G	R			
											A:13.6%			
11355C	ORF1a: A3697V	nsp6:A128V		C					C	C				Y
														T:35.8%
11516G	ORF1a: V3751I	nsp6:V182I		G					G	G	R			
											A:31.4%			
17977C	ORF1b: L1504F	nsp13:L581F		C		Y			C	C				
						T:21.6%								
19284T	ORF1b: D1939E	nsp14:D415E		T					T	T				W
														A:34.8%
26536A	M: N5S	M:N5S		A					A	A			R	
													G:31.5%	
PANGO			-	BA.1.1	BA.1.1	BA.1.1	BA.1.1	BA.1.1	BA.1.18	BA.1.1	BA.1.1	BA.1.1	BA.1.1	BA.1.1

Supplementary Table 4 Omicron BA.2 variants detected in nasal wash fluid samples from hamsters after second inoculation

Variant			Inoculum		Group/Animal No.				Inoculum		Group/Animal No.			
			1st	2nd	DMEM-BA.2				1st	2nd	BA.1-BA.2			
Nuc no. in Wuhan	AA substitution	protein	DMEM	TY40-385	#9	#10	#11	#12	TY38-873	TY40-385	#25	#26	#27	#28
10512A	ORF1a: D3416G	3CLpro:D153G	-	A				R	A	A				
								G:10.3%						
28471C	Silent	N		C					C	C				Y
														T:10.7%
PANGO				BA.2	BA.2	BA.2	BA.2	BA.2	BA.1.18	BA.2	BA.2	BA.2	BA.2	BA.2

Supplementary Table 5 Omicron BA.2.3 variants detected in nasal wash fluid samples from hamsters after second inoculation

Variant			Inoculum		Group/Animal No.				Inoculum		Group/Animal No.			
			1st	2nd	DMEM-BA.2.3				1st	2nd	BA.1-BA.2.3			
Nuc no. in Wuhan	AA substitution	protein	DMEM	TY40-816	#13	#14	#15	#16	TY38-873	TY40-816	#29	#30*	#31	#32
3784C	Silent	nsp3		C					C	C	Y<br>T:18.3%			
7093C	Silent	nsp3		C					C	C				Y<br>T:13.0%
7317T	ORF1a: F2351S	nsp3: F1533S		T					T	T	Y<br>C:44.6%			
11750C	ORF1a: L3829F	nsp6:L260F		C			Y<br>T:21.4%		C	C				
12060A	ORF1a: E3932A	nsp7:G73A		A					A	A				M<br>C:37.3%
26198C	ORF3a: T269M	ORF3a:T269M		C					C	C				Y<br>T:14.2%
PANGO				BA.2.3	BA.2.3	BA.2.3	BA.2.3	BA.2.3	BA.1.18	BA.2.3	BA.2.3	BA.2.3	BA.2.3	BA.2.3

*Animal #30 had other mutations. See Supplementary Table 6

Supplementary Table 6 Omicron BA.2.3 variants detected in a nasal wash fluid sample from the animal #30 after reinfection

			Inoculum		Group/Animal No.
Variant			1st	2nd	BA.1-BA.2.3
Nuc no. in Wuhan	AA substitution	Protein	TY38-873	TY40-816	#30
2086G	ORF1a: Q607H	nsp2: Q427H	G	G	K<br>T:10.9%
4250T	ORF1a: Y1329H	nsp3: Y511H	T	T	Y<br>C:15.2%
5514T	ORF1a: V1750A	nsp3: V932A	T	T	Y<br>C:15.9%
5763G	ORF1a: C1833F	nsp3: C1015F	G	G	K<br>T:10.9%
8600G	ORF1a: V2779L	nsp4: V16L	G	G	S<br>C:12.8%
12054T	ORF1a: C3931fs	nsp7: C72fs	T	T	del<br>10.3%
12467G	ORF1a: A4068T	nsp8: A126T	G	G	R<br>A:10.8%
12708C	ORF1a: A4148V	nsp9: A8V	C	C	Y<br>T:10.1%
14460T	ORF1b: F331L	nsp12: F340L	T	T	K<br>G:22.3%
18352T	ORF1b: L1629del, P1630del	nsp14: L105del, P106del	T	T	del<br>13.4%
18353T			T	T	del<br>13.4%
18354A			A	A	del<br>13.4%
18355C			C	C	del<br>13.4%
18356C			C	C	del<br>13.4%
18357T			T	T	del<br>13.4%
18786T	Silent	nsp14	T	T	Y<br>C:10.3%
21575C	S: L5F	S: L5F	C	C	Y<br>T:11.4%
23766C	S: S735L	S: S735L	C	C	Y<br>T:11.4%
25613C	ORF3a: S74F	ORF3a: S74F	C	C	Y<br>T:13.0%
25714C	ORF3a: L108F	ORF3a: L108F	C	C	Y<br>T:12.3%
26355T	Silent	E	T	T	Y<br>C:13.2%
26413T	E: Y57H	E: Y57H	T	T	Y<br>C:12.8%
28864A	Silent	N	A	A	W<br>T:18.9%
29409C	N: T379N	N: T379N	C	C	M<br>A:16.1%
29708C	Silent	3'UTR	C	C	Y<br>T:11.2%
PANGO			BA.1.18	BA.2.3	BA.2.3
